# Mussel Inspired Polynorepinephrine Functionalized Electrospun Polycaprolactone Microfibers for Muscle Regeneration

**DOI:** 10.1038/s41598-017-08572-z

**Published:** 2017-08-15

**Authors:** Ying Liu, Guoqiang Zhou, Zhu Liu, Mengyu Guo, Xiumei Jiang, Mehmet Berat Taskin, Zhongyang Zhang, Jing Liu, Jinglong Tang, Ru Bai, Flemming Besenbacher, Menglin Chen, Chunying Chen

**Affiliations:** 1 CAS Key Laboratory for Biomedical Effects of Nanomaterials and Nanosafety and CAS Center for Excellence in Nanoscience, National Center for Nanoscience and Technology of China (NCNST), Beijing, 100190 China; 2 Interdisciplinary Nanoscience Center (iNANO), Aarhus University, DK-8000 Aarhus C, Denmark; 3 Key Laboratory of Medicinal Chemistry and Molecular Diagnosis of Ministry of Education, College of Chemistry and Environmental Science, Hebei University, Baoding, 071002 China

## Abstract

Electrospun scaffolds with excellent mechanical properties, high specific surface area and a commendable porous network are widely used in tissue engineering. Improving the hydrophilicity and cell adhesion of hydrophobic substrates is the key point to enhance the effectiveness of electrospun scaffolds. In this study, polycaprolactone (PCL) fibrous membranes with appropriate diameter were selected and coated by mussel-inspired poly norepinephrine (pNE). And norepinephrine is a catecholamine functioning as a hormone and neurotransmitter in the human brain. The membrane with smaller diameter fibers, a relative larger specific surface area and the suitable pNE functionalization provided more suitable microenvironment for cell adhesion and proliferation both *in vitro* and *in vivo*. The regenerated muscle layer can be integrated well with fibrous membranes and surrounding tissues at the impaired site and thus the mechanical strength reached the value of native tissue. The underlying molecular mechanism is mediated via inhibiting myostatin expression by PI3K/AKT/mTOR hypertrophy pathway. The properly functionalized fibrous membranes hold the potential for repairing muscle injuries. Our current work also provides an insight for rational design and development of better tissue engineering materials for skeletal muscle regeneration.

## Introduction

Tissue engineering provides a potential treatment for a wide range of diseases. The musculoskeletal system, including bone^1, 2^, tendon/ligament^3^ and muscles^4, 5^, has become the major target for tissue engineering. The efficacy of musculoskeletal tissue engineering relies highly on the scaffolds, which should essentially support cells to form neotissues at the target sites. The scaffold used for musculoskeletal tissue engineering should be able to withstand certain tensile force, reduce cicatrix formation and provide suitable microenvironment for cell growth and proliferation. And the degradation cycle of the polymer scaffold should meet the muscular remodeling period.

Different fabrication methods have been used to prepare biomaterials with various properties for the rebuilding of the musculoskeletal system according to the specfic property of target tissues. Electrospinning has attracted wide attention as an excellent way to prepare continuous non-woven fabric fibers^6^ in nano- and micro-scale with high porosity, large surface area, and controllable mechanical properties. Electrospun membranes highly imitate natural extracellular matrix (ECM) and thus have been widely used for various biomedical applications, such as tissue engineering, drug delivery and regenerative medicine^7, 8^. Soft electrospun polymer membrane is recognized as an ideal material in muscle injure recovery for its good cytocompatibility, biodegradability and excellent mechanical properties^9^. Moreover, its ECM-like structure improves cell attachment on the membrane and provides better microenvironment for cell growth. It has been reported that biodegradable polycaprolactone (PCL)^10^, poly L-lactic acid (PLA), polyglycolic acid (PGA), poly L-lacticacid/polyglycolic acid (PLGA) copolymer were used as musculoskeletal tissue engineering scaffold materials^11–16^. PCL has good biocompatibility and low immunogenicity in body, which can slowly degrade in the body into non-toxic metabolic products. The degradation time frame of PCL fibers was even more than 2 years^17^, which was suitable for the metabolism and regeneration time of muscles. Muscle stem cells were attracted to the injured area and become muscle cells during the regeneration progress of muscles^18^.

In addition to biocompatibility, the physiological compatibility between cells and the biomaterial surface is critical in order to support tissue reconstruction of skeletal muscle in the injured region. Up to now, various biological macromolecules including chitosan, gelatin, collagen^19^, laminin and growth factor have been used to modify the scaffold surface *via* EDAC coupling, electrostatic layer-by-layer deposition, self-assembly grafting and so on. Since its discovery in 2007^20^, poly dopamine (pDA) as mussel-inspired substrate-independent coating method has been applied on a range of biomaterials (microfluidics, catalysts, bio-mineralization^21^, surface modifications^22^, sensitive determination^23^ and neural interfaces). Dopamine (DA) and norepinephrine (NE) as catechol molecules can enhance surface affinity *via* self-polymerization on virtually any surfaces in alkaline environment. When compared to pDA, the polymerization process of pNE is much more controllable, generating a more uniform and thinner coating, so that the pNE coating could be applied to nano/submicro-featured scaffold surface^24^. Although pDA has been applied as biomaterials in many studies, pNE coating has only been recently applied for neural interfacing, where the introduced catechol groups from pNE could not only anchor collagen to enhance cell adhesion but also localize nerve growth factor to promote neuron-like differentiation^25^. The pNE functionalized biomaterials have not been investigated as tissue engineering scaffolds *in vivo*. Besides, previous researchers have explored the effect of fiber diameter on cell adhesion and proliferation which shows that fibrous membranes with smaller diameters may generally result in better cell migration, spread, signaling and replication^26^. Therefore, the fiber diameter of electrospun membranes and their surface affinity play important roles in tissue engineering.

**Figure 1:**
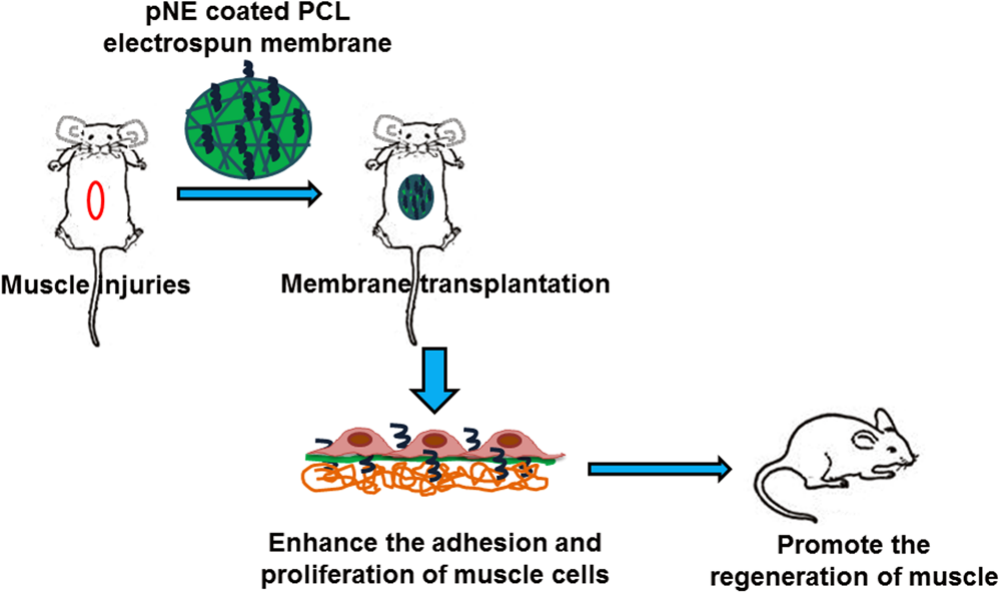
Scheme of pNE coated PCL electrospun fibrous membranes and their effect on muscle regeneration *in vitro* and *in vivo*.

Herein, in order to make scaffold withstand tensile force, reduce cicatrix formation and provide suitable microenvironment for cell growth and proliferation, we designed and fabricated PCL fibrous membranes composed of different fiber diameters (2 μm and 10 μm) *via* electrospinning, named 2 P and 10 P membrane respectively. Then, the membranes were coated by pNE *via* self-polymerization. The collective impacts of surface affinity and fiber diameter on the attachment and proliferation of muscle cells were investigated. These different PCL fibrous membranes with or without pNE coating were used as scaffolds for further skeletal muscle regeneration investigation. Figure 1 shows the experiment design including 1) fabrication of pNE coated PCL fibrous membranes, 2) pNE coated PCL fibrous membranes regulating the proliferation and morphology of muscle cells *in vitro*, 3) pNE coated PCL fibrous membranes promoting muscle regeneration *in vivo*.

## Results

### Thinner electrospun PCL fibers were coated by pNE more effectively

PCL fibrous membranes composed of randomly aligned fibers with diameters of 2 μm (2 P) and 10 μm (10 P) were fabricated *via* electrospinning. Then the membranes were coated by pNE and named as 2 PP and 10 PP membranes, respectively (Figure S1).

**Figure 2:**
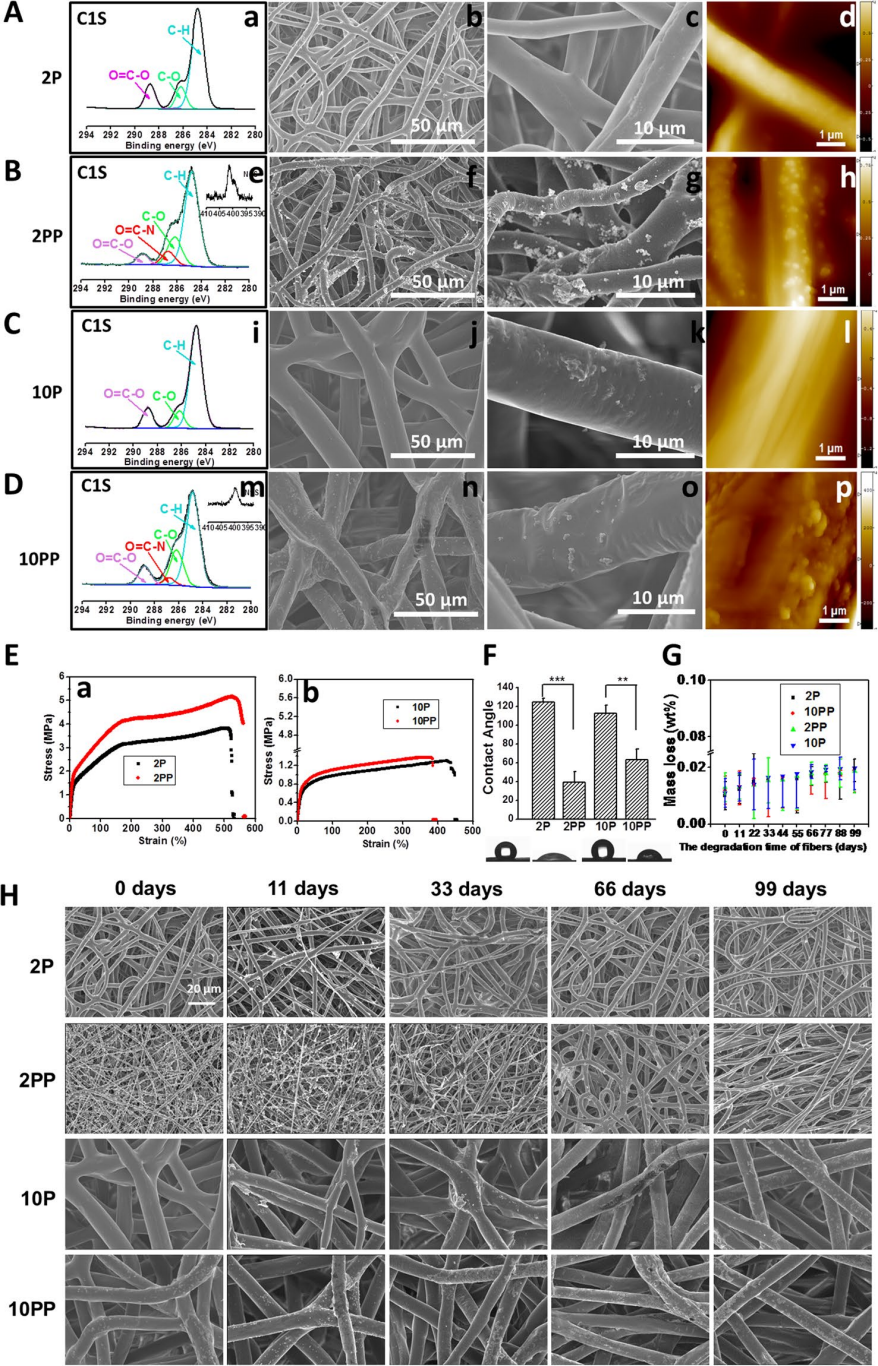
Characterization of PCL fibrous membranes. (**A**–**D**) XPS spectra (a,e,i,m), SEM (b,c,f,g,j,k,n,o) and AFM images (d,h,l,p). A, PCL fibrous membrane with 2 μm in diameter (2 P). B, pNE coated PCL fibrous membrane with 2 μm in diameter (2 PP). C, PCL fibrous membrane with 10 μm in diameter (10 P). D, pNE coated PCL fibrous membrane with 10 μm in diameter (10 PP). (E-H) The mechanical property, wettability and degradation *in vitro* of PCL fibrous membranes. E, The stress-strain curves of PCL fibrous membranes. F, Images and quantitative statistics of contact angle. G, Mass loss of PCL fibrous membranes after cultured in PBS at 37 °C for 11, 33, 66 and 99 days, respectively. H, SEM images of PCL fibrous membranes after cultured in PBS at 37 °C for 11, 33, 66 and 99 days.

As shown in Fig. 2, the surface roughness of fibers before and after coated by pNE was observed using scanning electron microscope (SEM) and atomic force microscope (AFM). And we found that pNE resulted in a rougher surface on fibers (Fig. 1h and p). For short coating times of pNE on fibers, a smooth surface on fibers was observed, whereas longer coating time of pNE resulted in a rougher surface on fibers (Figure S2). The elemental composition of the fibrous surfaces was characterized using XPS analysis (a, e, i, m). In XPS spectrum, nitrogen peak (398 eV) can be used to distinguish pNE coating on the PCL fibers. Oxygen, carbon and nitrogen peaks of PCL fibrous membranes with pNE coating (2 PP and 10 PP) were all apparent, while only oxygen and carbon peaks were found for those without pNE (2 P and 10 P). The intensity of the nitrogen peak of thinner PCL fibrous membranes with pNE coating (2 PP) was nearly twice higher than that of 10 PP (e, m) (Table 1). Similar results were observed using scanning electron microscopy (SEM) and atomic force microscopy (AFM) to characterize the fiber surface morphology changes of pNE coated PCL fibrous membranes. Thinner PCL fibers (2 μm, f, g, h) were significantly easier to be coated by pNE than thicker ones (10 μm, n, o, p). The coating amount of pNE on the thinner PCL fibers was significantly more than that on the thicker ones. A possible reason is that the fibers have larger surface area because of the decreased diameter.

**Table 1:** Elemental composition of fibrous membrane surfaces.

Elements	C (%)	O (%)	N (%)	N/C (%)
2 P	78.886	21.114	0	0
2 PP	74.384	21.252	4.364	5.87
10 P	80.923	19.077	0	0
10 PP	75.111	22.68	2.21	2.94

The mechanical properties of the fibrous membranes were shown in the stress-strain curves (Fig. 2E). Membranes with smaller fibers have higher strength than those with larger fibers, and pNE coating further increased the strength, especially 2PP.

The wettabilities of these membranes were investigated by measuring water contact angles, providing intuitive information about the influence of pNE coating on the fibers surface hydrophilicity. The water contact angles of 2 PP and 10 PP membrane (with pNE coating) were 121 ± 1° and 118 ± 1°, while that of 2 P and 10 P membrane (without pNE coating) were 37 ± 2° and 61 ± 1° (Fig. 2F), respectively. Hence, pNE coating changed the inherent hydrophobicity of fibrous membranes and increased their wettability significantly. Moreover, the water contact angle of 2 PP membrane was lower than that of 10 PP membrane, which indicates that the fibrous membrane with smaller diameter was more hydrophilic after pNE coating. Meanwhile, there was also a possibility that the difference in wettability of fibrous membrane was related to the amount of pNE coated on the fibers.

Degradation of the fibrous membranes *in vitro* was monitored for 99 days, where no obvious mass loss or morphology changes were observed (Fig. 2G,H).

### Thinner pNE Functionalized membranes were effective for repairing muscle injury

**Figure 3:**
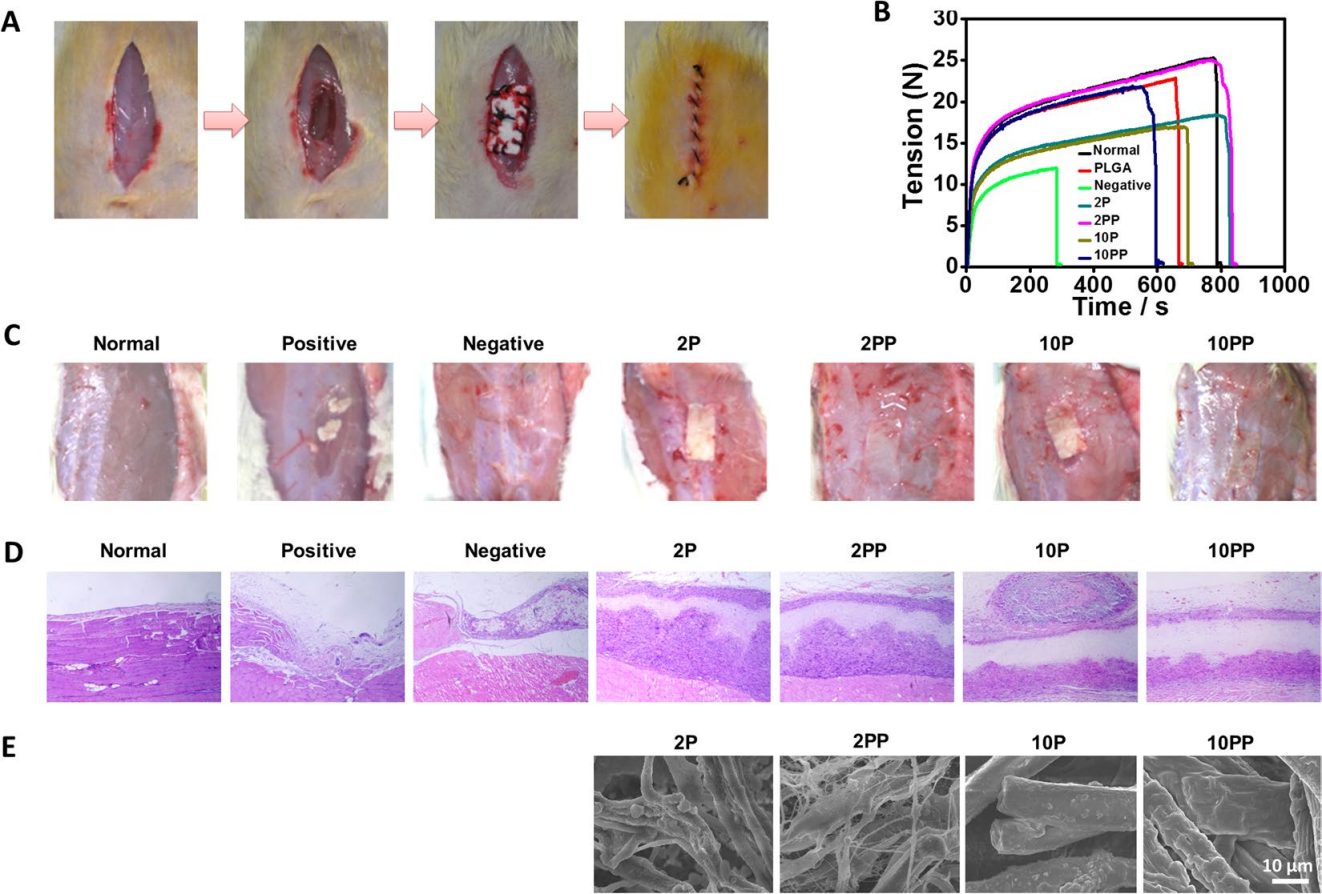
Functionalized electrospun PCL memberanes were effective for the regenerating muscle injury. (**A**) Diagram of PCL fibrous membranes implantation *in vivo*. After implantation into rat muscles for 40 days, mechanical properties of PCL fibrous membranes (**B**), visible morphology (**C**) and histological observations (**D**) of muscles and SEM images of PCL fibrous membranes (**E**) were observed.

Rat muscle injury model was used for implanting PCL fibrous membranes to repair impaired musculus rectus abdominis (Fig. 3A). At day 40 after implantation, PCL fibrous membranes together with the integrated tissue were removed and analyzed. As shown in Fig. 3B, the tensile strength of 2 PP group was much higher than the other groups which reached similar value of the normal muscle tissue. In consistence, the regenerated muscle layer integrated well with fibrous membranes and surrounding tissues at the impaired site (Fig. 3C), which could be presented clearly by using histological observations (Fig. 3D).

No membranes were implanted on the muscle defects in negative group, whereas the membrane was composed of PLGA which was degraded rapidly *in vivo* used for comparison. There were still obvious muscle defects covered by a thin layer of fibrous connective tissue and inflammatory cells in negative group and PLGA group. All functionalized PCL fibrous membranes were effective for repairing muscle injury, though serious inflammation was found in 10 PP group. In addition, more cells were found to migrate into the thinner fibrous membranes (2 P and 2 PP) than those on thicker ones. The fibrous membranes with a small diameter were more suitable to the formation of muscle. Since pNE coating inverted hydrophobic fibers to hydrophilic ones, it can provide better microenvironment for cell adhesion and proliferation.

### Thinner pNE functionalized PCL microfibers (2PP) were more effective for muscle cell proliferation and adhesion

**Figure 4:**
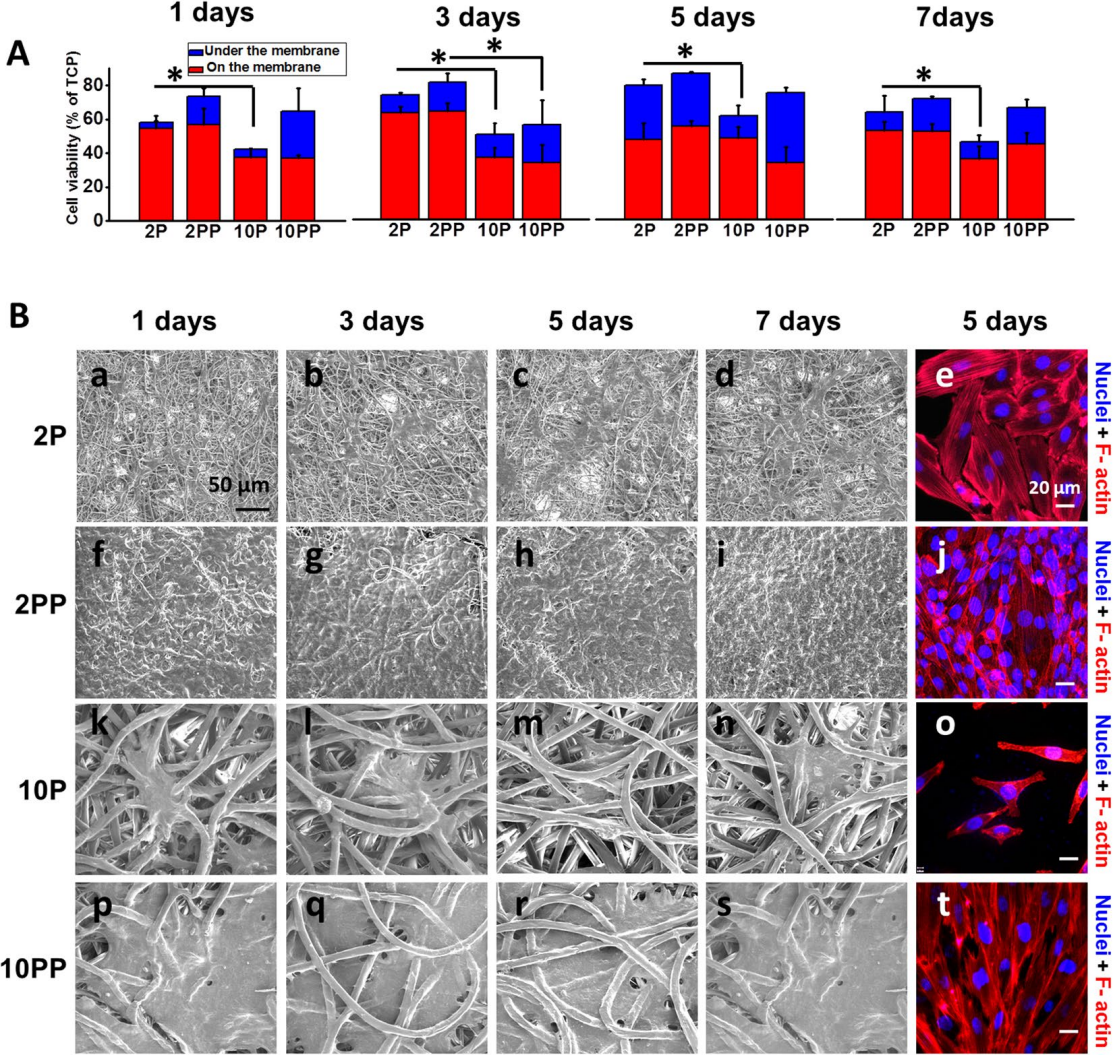
Thinner functionalized electrospun PCL microfibers (2 PP) were more effective for the muscle cell proliferation and adhesion. (**A**) After cultured on PCL fibrous membranes for 1, 3, 5 and 7 days, cell proliferation was detected using CCK-8. (**B**) ESEM and fluorescent images of cells on PCL fibrous membranes.

To assess the cell proliferation and adhesion of PCL fibrous membranes *in vitro*, L6 skeletal muscle cells were cultured on PCL fibrous membranes. The *in vitro* proliferation of cells on PCL fibrous membranes was measured by CCK-8 kit. pNE improves the proliferation of cells on PCL fibrous membranes. The cell viability on PCL fibrous membrane with diameter of about 2 μm (2 P and 2 PP) is better than 10 μm (10 P and 10 PP) (Fig. 4A). More cells grew on the thinner pNE coated PCL fibrous membranes (2 PP) than other membranes. The extension, proliferation and adhesion of skeletal muscle cells on the fibrous membrane of different diameters were also observed on day 1, 3, 5 and 7. Cells in the 2 PP and the 10 PP membranes were more elongated than those in 2 P and 10 P, and cells in 2 P and 2 PP spread much better than those in 10 P and 10 PP (Fig. 4B). This is attributed to the higher surface area in the thinner fibers and pNE coating collectively enhanced the ECM proteins deposition on the fibers, which promoted cell adhesion and proliferation.

**Figure 5:**
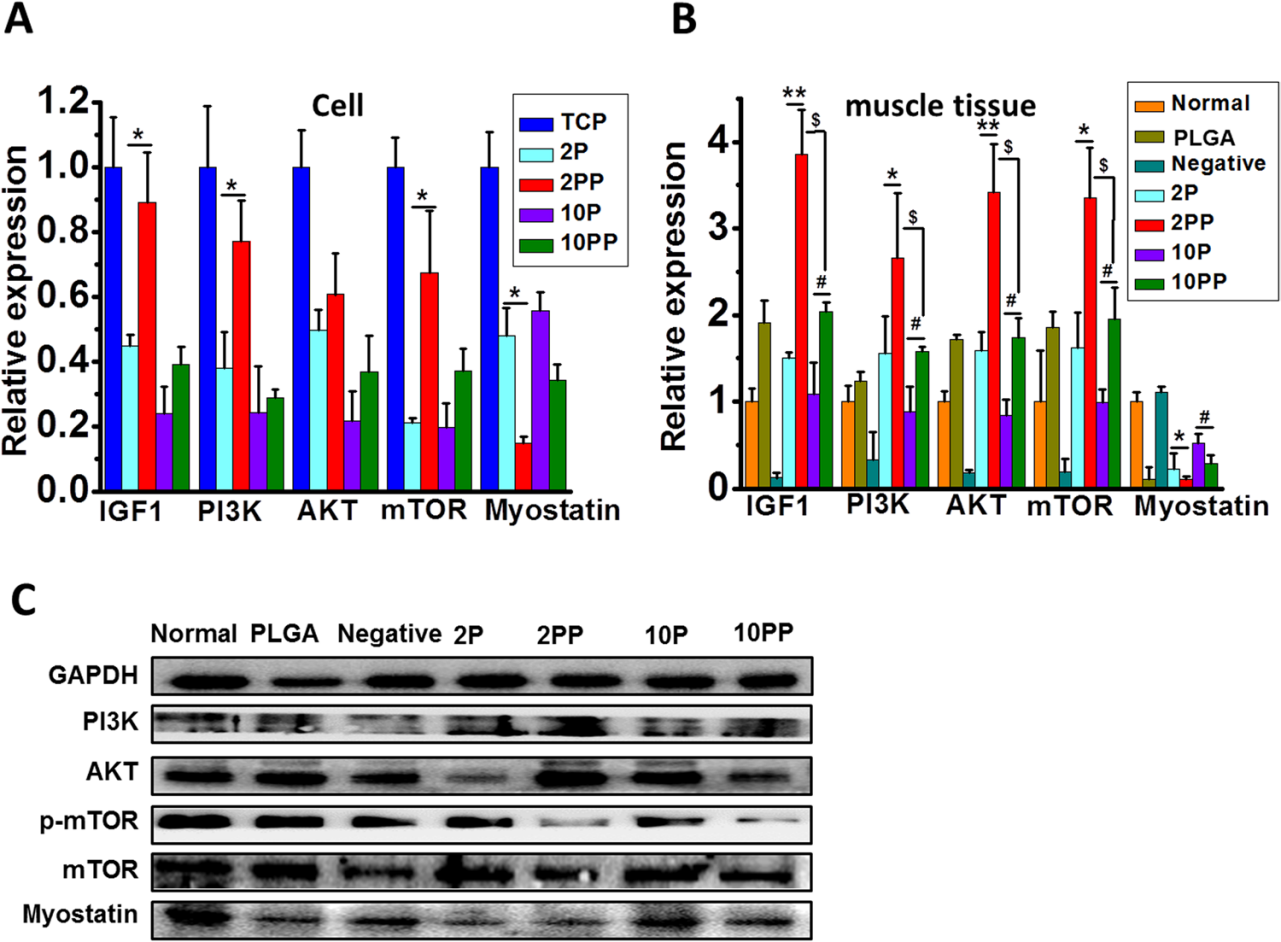
Thinner functionalized electrospun PCL microfibers (2 PP) were more effective for the muscle cell growth and differentiation. (**A**,**B**) Relative expression of IGF1, PI3K, AKT, mTOR and myostatin of cells and muscle tissues. C, Representative western blotting of PI3K, AKT and myostatin of muscle tissues from *in vivo* experiments. ^*, #, $^ *p* < 0.05 and **p < 0.01 as indicated.

Various proteins are closely related to the reduction of muscle mass and hypertrophy. Among them, myostatin is a well-known negative regulator of skeletal muscles and plays a key role in the development and the maintenance of muscles. Myostatin, known as an inhibiting gene of skeletal muscle proliferation, would increase with muscle atrophy or injuries^27^. As shown in Fig. 5, the myostatin mRNA and protein expression was used to evaluate the proliferation of myoblasts. The myostatin mRNA expression significantly decreased in 2 P and 2 PP than 10 P and 10 PP *in vivo* and *in vitro*, where expression in 2 PP and 10 PP are higher than that in 2 P and 10 P, respectively (*p* < 0.05). The reduced myostatin expression in 2PP group indicates that pNE further suppressed myostatin expression, and the reduction led to the increased myocyte proliferation in the muscle. That is to say, thinner PCL microfibers coated by pNE (2PP) induced an increase in myocyte proliferation by suppressing myostatin expression.

Previous research found that myostatin signaling reversed the insulin-like growth factor 1/phosphatidyl inositol 3-kinase/protein kinase B (IGF-1/PI3K/Akt) hypertrophy pathway by inhibiting AKT, allowing for increased expression of atrophy-related genes^27, 28^. The mammalian target of rapamycin (mTOR) is an important downstream mediator in the PI3K/Akt signaling pathway, which plays a critical role in regulating cell proliferation, survival, mobility and angiogenesis^29^. In the present study, we found that the mRNA expression of IGF-1, PI3K, AKT and mTOR significantly increased in 2 PP than 10 PP *in vivo* and *in vitro* (*p* < 0.05). The result indicates that thinner PCL microfibers coated by pNE (2PP) inhibit myostatin expression by PI3K/AKT/mTOR hypertrophy pathway, leading to the increased myocyte proliferation in the muscle.

### Thinner pNE functionalized membranes degraded faster *in vivo* with no toxicity identified

In the design and development of an implant material, *in vivo* degradation behavior is very important. As a solid implant material, slow degradation rate would be ideal which was consistent with the time span for musclar reconstruction. PCL fibers were all still present with the fibrous structure at day 40 after implantion. Degradation of PCL fibers coated by pNE (2 PP and 10 PP) *in vivo* were quicker than that without pNE coating (2 P and 10 P) (Fig. 3E). Indicating pNE coating accelerated the degradation of PCL fibers, especially the thinner fibers. The *in vitro* degradation process is based on the hydrolytic reaction and mostly follows the heterogeneous degradation mechanism. No obvious morphology wastage (Fig. 2H, Figure S3) and weight loss (Fig. 2G) were observed until PCL fibrous membranes were cultured for 99 days in PBS at 37 °C.

Even though pNE has been used for functionalizing scaffold surfaces, its *in vivo* toxicology is still unknown. Here, comprehensive and meticulous investigations on toxicology were carried out that implys all the fibrous membranes were quite safe and biocompatible. In all defected groups, there were no differences in the body weight compared with normal control group. There was no change in heart, liver, spleen, lung and kidney coefficients in normal and all the fibrous membrane groups (Table S1), which was further confirmed by the histological examination (Figure S2). No differences were found by the complete blood counts (Table S3 and 4) and blood coagulation factor (Table S2) analysis. In blood biochemical analyses, serum blood urea nitrogen (BUN) and creatinine (Cr) were used to analyze the cause of acute renal injury or dehydration, alanine transaminase (ALT) and aspartate transaminase (AST) were used to evaluate hepatic damage (Table S5). Similarily, there are no significant difference between normal control and fibrous membrane treated mice, which suggest that fibrous membrane had no toxicity to liver and kidney after 40 days implantation (*p* > 0.05).

## Discussion

Advances in biomaterial fabrication techniques have broadened their application in biomedical engineering. The success of biomaterials replies on their abilities to modulate cell and tissue responses, such as cell adhesion, as well as induction of tissue repair processes^30^. In this article, traditional electrospinning technique was adopted to prepare fibrous membranes with different diameters, and then the membranes were coated by pNE *via* self-polymerization. By analyzing the chemical components of fiber surface and measuring the contact angle, it can be proved that the fibers were successful decorated by pNE, which highly improved affinity of the fibrous membrane. In addition, we also found that the fibrous membrane with smaller diameter has a relatively larger specific surface area. *In vitro* cell experiments demonstrated that pNE coating and fiber diameter highly affected the adhesion and proliferation of skeletal muscle cells on the PCL electrospun membranes. pNE coated membranes with smaller diameter fibers (2 μm) support the best adhesion and proliferation of cells, which may result from the highly improved wettability and larger specific surface area. The underlying molecular mechanism is mediated *via* inhibiting myostatin expression by IGF-1/PI3K/AKT hypertrophy pathway, leading to the increased myocyte proliferation in the muscle. According to the HE analysis of muscles and main organs after implantation *in vivo* for 40 days, there is no obvious toxicity observed. Furthermore, the regenerated muscle layer integrated well with fibrous membranes and surrounding tissues at the impaired site and the mechanical strength reached the value of native tissue. Therefore, we can deduce that functionalized fibrous membranes are promising for repairing skeletal muscle injuries.

Muscle tissue engineering aims to reconstruct skeletal muscle loss resulting from injury, congenital defects, or tumor ablations^31^. Muscle cells possess relatively limited ability to regenerate under *in vivo* conditions^32^. The success of muscle tissue engineering depends highly on the ability to modulate cell adhesion, and proliferation, induction of repair and immune processes^33, 34^. Inspired by mussel adhesiveness to surfaces even in wet conditions, scientists discovered that dopamine (DA) undergoes self-polymerization at alkaline conditions. This reaction comprising a relatively facile procedure provides a universal coating method regardless of chemical and physical properties of the substrates. Furthermore, this polymerized layer is enriched with catechol groups that enable immobilization of primary amine or thiol-based biomolecules via a simple dipping process. Although pDA has, in many instances, shown to be able to facilitate adhesion of cells and biomolecules, and has therefore been extensively explored for tissue engineering applications^35^, the pDA coating on nano/microscale topography is hampered by their quick aggregation. Recently, the biocompatibility analysis and cell proliferation results suggest that the PNE coating, with slower polymerization kinetics compared to pDA, is more biocompatible with both hiPS-MSCs and hMSCs than the PDA coating^36^. Here we have for the first time applied pNE to coat PCL fibers for muscle tissue engineering, and found that the muscle cells adhesion and proliferation were enhanced with promising tissue restoration on pNE/PCL fibers compared to the unmodified couterparts. This is consistent with a recent study of Ku *et al*., which demonstrated that myogenic protein expression and myoblast fusion were upregulated on pDA/PCL nanofibers^37^.

Mussel inspired coating is relatively nascent in muscle tissue engineering, and the cell-material interaction mechanism is unclear. As the first study using pNE functionalization in muscle tissue engineering, our research suggests that muscle cells interact highly with pNE modified surfaces with no evidence of *in vivo* cytotoxicity. With further validations in polymeric biomaterial design, pNE may be a promising tool for altering the surface of various materials for muscle tissue engineering. Our current work provides an insight for rational design and development of better tissue engineering materials for skeletal muscle regeneration.

## Methods

### Preparation of PCL electrospun fibers with different diameters

Polycaprolactone (PCL) fibrous membranes were prepared using electro spinning. PCL (Mw = 80,000; Sigma, St. Louis, MO, USA) was dissolved in 17.5% (W/V) trichloromethane (Sigma, St. Louis, MO, USA) and stirred overnight at RT. The polymer solution was electrospun on the collector with single-layer tin-foil (15 kv, 20 G cut-ended needle, 10 mL syringe, 3.6 mL/h speed, 15 cm distance) to obtain 10 μm fibers. PCL was also dissolved in trichloromethane/ethanol (7:3, V/V) at the concentration of 15% (W/V) by stirring overnight at RT. The final solution was electrospun on the collector with single-layer tin-foil (20 kv, 20 G needle, 10 Ml syringe, 10 mL/h speed, 20 cm distance) to obtain 2 μm fibers. Both the two kinds of membranes were lyophilized overnight.

### Polynorepinephrine (pNE) coating

For coating pNE on the fibers, PCL fibrous membranes were immersed in 2 mg/mL norepinephrine (Sigma, St. Louis, MO, USA) solution in pH = 8.5 Tris-HCl buffer at RT for 15 h. Followed by three cycles of washing steps with water to fully remove the residual liquid, then the samples were dried at RT and stored at 4 °C.

### Characterization of engineered fibrous membranes

#### X-ray photoelectron spectroscopy (XPS)

The surface chemical composition of the developed scaffold was analyzed by XPS and XPS valence band spectra were obtained on an ESCALAB 250Xi instrument (Thermo Scientific). The depth of analysis was 50–100 Å, and the surface area analyzed was 2 mm × 3 mm for each sample.

#### Scanning electron microscopy (SEM)

The surface morphologies of 2 P, 2 PP, 10 P and 10 PP fibrous membranes were examined with a high-resolution SEM (Hitachi S4800, Japan). The fibrous membranes were placed directly into the SEM chamber; all the images were captured using a secondary electron detector with an acceleration voltage of 5 kV.

#### Atomic force microscopy (AFM)

AFM is applied to study the adsorption. The force-volume mode is used to record the force-deformation curves of the adsorbed molecules on the fiber surface.

#### Mechanical property

Mechanical stability of scaffold was examined by using a universal tensile tester (Instron-336, USA) with load cell capacity of 10 N. Six parallels were set for each group (2 P, 2PP, 10 P, 10PP) and each film was measured for thickness. For testing, long strips with dimension of 2 cm × 8 cm were properly fixed in the grips and permitted to elongate at an extension speed of 30 mm/min. Stretched the film until it was pulled.

#### Contact Angle (CA)

In order to determine the hydrophilicity of pNE functionalized PCL fibrous membranes, wettability was tested by means of a Krüss Drop Shape Analysis System (DSA100, Germany). A water droplet of 1 mL was dropped on the surface of fibrous membranes, and the CA values were recorded by continuous shooting mode with an interval of 5 ps^−1^ for 15 s. Each sample was measured three times at different positions.

#### Transmission electron microscopy (TEM)

The interior structures of the fibrous membranes were detected using TEM (Hitachi, HT7700) at 200 kV. All the samples were prepared by electrospinning membranes directly on carbon coated copper grids.

### Mass loss test

The mass loss of electrospun membranes was studied under physiological condition (PBS, pH = 7.4, 37 °C). After weighed (W0), fibrous membranes were cultured in 20 ml PBS for more than three months. At 0, 11^th^, 22^th^, 33^th^, 44^th^, 55^th^, 66^th^, 77^th^, 88^th^ and 99^th^ day, the fibrous membranes were taken out, freeze-dried overnight and weighed (W1). This conversion is calculated in terms of mass loss as: Mass loss = (W0 − W1)/W0 × 100%.

### Cell viability

Skeletal muscle cell line L6 was purchased from the American Type Culture Collection (Rockville, Maryland, USA) and cultured in DMEM-L medium (WISENT Inc., Quebec, Canada) supplemented with 10% fetal bovine serum (FBS; Gibco, Langley, Oklahoma, USA), 2 mM L-glutamine, 20 mM HEPES, 100 U/mL penicillin and 1 mg/mL streptomycin (WISENT Inc., Quebec, Canada). Cells were maintained under standard cell culture conditions (37 °C in a humidified atmosphere of 5% CO_2_).

For experiments of cell proliferation and adhesion, 4 groups of fibrous membranes of 12 mm Ø (about 1.1 cm^2^) were punched out using micro-punches (CaronnoPortusella, Milan, Italy), and placed on the bottom of a sterile standard 48-well plate. For cell proliferation study, L6 cells were respectively seeded onto each membrane at a density of 3 × 10^5^, 2 × 10^5^, 1.5 × 10^5^, 1 × 10^5^ cells for 1 d, 3 d, 5 d and 7 d. In parallel, cells seeded on a 48-well cell culture plate served as control.

Cell viability under and on the membrane were measured using Cell Count Kit-8 (CCK-8) (Dojindo Laboratories, Japan). The CCK-8 contains WST-8 [2-(2-methoxy-4-nitrophenyl)- 3-(4-nitrophenyl)-5-(2,4-disulfophenyl)-2H-tetrazolium, monosodium salt] which becomes a water-soluble formazan dye upon reduction with the dehydrogenase in the mitochondria. Briefly, cells were incubated with 100 μL of culture medium in 96-multiwell plates. After treatment, the culture medium was discarded and 110 μL of fresh culture medium containing 10% CCK-8 was added to each well. After incubated for 2 h at 37 °C, the value of the absorbance at 450 nm was measured by using a microplate reader (TECAN, Durham, USA). The cell viability was calculated by the equal:

$${\rm{\eta }}\,=\,({{\rm{OD}}}_{{\rm{test}}}-{{\rm{OD}}}_{{\rm{blank}}})/({{\rm{OD}}}_{{\rm{control}}}-{{\rm{OD}}}_{{\rm{blank}}})\,\times \,100 \% .$$

### Cytoskeleton of skeletal muscle cells

1 × 10^5^ L6 cell were cultured on fibrous membranes for 7 d and fixed with 4% paraformaldehyde in PBS buffer (pH = 7.4). After washed three times with PBS buffer (pH = 7.4), the fibrous membranes were incubated with permeabilization buffer (0.5% Triton X-100) for 5 min. After washed other three times, the fibrous membranes were incubated with 100 nM Rhodamine-phalloidin (Invitrogen, USA) for 30 min and 100 nM Dihydrochloride (DAPI) for 20 min. Cytoskeleton of skeletal muscle cells was obtained by single photon laser confocal microscope (Zeiss, Germany).

### Environmental scanning electron microscope (ESEM)

The environmental scanning electron microscope (ESEM) can work at room conditions and allows to observe wet and oily materials without any need of dehydration and of making the sample electro-conductive. The fibrous membranes were fixed in 2.5% glutaraldehyde overnight and the gradient dehydration by alcohol (30%, 50%, 70%, 85%, 95% and 100%). After dried at RT for 3 days, the images were obtained using ESEM (Quanta200, FEI, USA).

### *In vivo* experiments

All animals were provided by the Laboratory of Experimental Animals of the Chinese Academy of Medical Sciences and housed in a temperature-controlled, ventilated and standardized disinfected animal room. Animals were allowed to acclimatize, without handling, for a minimum of 1 week before the start of experiments. All animal experiments were conducted using protocols approved by the Institutional Animal Care and Use Committee at the Institute of Tumors of the Chinese Academy of Medical Sciences.

Six week Sprague-Dawley female rats (160–170 g) were used as recipients in biocompatibility evaluation of electrospun membranes *in vivo*. The total of 35 rats was divided into 7 groups: Normal group (n = 5), Negative group (n = 5), PLGA group (n = 5), 2 P group (n = 5), 2 PP group (n = 5), 10 P group (n = 5), 10 PP group (n = 5). All rats were sacrificed 40 days after the operation. Before treatment, a transperitoneal approach was performed to expose rat’s musculus rectus abdominis after anesthesia. 2 cm^2^ of the musculus rectus abdominis was taken off and peritoneum wasn’t penetrated.

The impaired musculus rectus abdominis was covered and stitched interruptedly with electrospun fibrous membrane. The area of each membrane used was about 6 cm^2^ (length was 3 cm and width was 2 cm). Before implantation, the membranes were sterilized use ultraviolet germicidal irradiation and sealed. Rats of negative control group were not blanketed with any anti-adhesion membranes. And then, the abdominal cavity was closed using 1–0 silk suture.

### Hematoxylin and eosin staining analysis

The hearts, livers, spleens, lungs and kidneys were fixed overnight in 10% formalin neutral buffer, dehydrated using a series of graded ethanol solutions and embedded in paraffin. Baseline histological slides with 4–5 mm sections were stained with hematoxylin/eosin (HE) and dehydrated through a graded series of ethanol solutions ranging from 75 to 100%. A well-trained pathologist then examined the slides blindly. Histological observations and photomicrography were performed using a light microscope (Life TechnologyTM).

### Quantitative real-time polymerase chain reaction (qRT-PCR)

Frozen muscle tissues were immersed in liquid nitrogen followed by grinding to a fine powder in a pre-chilled mortar and pestle. Total RNA was extracted from muscle tissues and L6 cells cultured on four kinds of fibrous membranes for 5 days using TRIZOL Reagent method (Invitrogen). Samples were suspended in 1 ml TRIZOL and disrupted using a dounce homogenizer with a tight-fitting pestle. The entire solution was subjected to 50 passes before spinning down at 9,000 × g for 5 min at 4 °C. The supernatant was saved and total RNA was extracted according to the manufacturer’s protocol and treated with RNase-free DNase (Promega) to eliminate genomic DNA. Relative genes expression was investigated using qRT-PCR and the 2(-delta Ct) method. PCR amplification was performed using the following conditions: 95 °C for 2 min, then 40 cycles of 95 °C for 15 sec and finally 60 °C for 1 min. Target gene expression was normalized to reference gene (GAPDH). The reactions were repeated three times. The primer sequences were as follows:

**Table:** 

	forward	reverse
IGF1	5′-CAGCATCGTGTGGCAGGAC-3′	5′-TCTTGGTCAGGTGGCGTAA-3′
PI3K	5′-AGGCTGTGATTGGGCGTA -3′	5′-AAGCAACCTCAAAGGGAAA-3′
AKT	5′-CTTGACATGAACCCAGGCAC-3′	5′-TTCAGCCCATCTTCTCCTGG-3′
mTOR	5′-GACGGTGTAGAACTTGGAGAA-3′	5′-TGAGATGTCGCTTGCTTGA-3′
Myostain	5′-CTACCACGGAAACAATCA TTA-3′	5′-AGCAACATTTGGGCTTTCCAT-3′
GAPDH	5′-AACTTTGGCATTGTGGAAGG -3′	5′-ACACATTGGGGGTAGGAACA-3′

### Western Blotting

The muscle tissues were homogenized with lysis buffer (50 mM Tris-HCl (pH 8.0), 150 mM NaCl, 10% glycerol, 1% Triton X-100, 1.5 mM MgCl, 1 mM ethylene glycol tetraacetic acid, 1 mM phenylmethylsulfonyl fluoride, 1 mM Na_2_VO_4_ and 100 mM NaF), and then centrifuged at 12,000 rpm for 10 minutes. The protein was separated on sodium dodecyl sulfate-polyacryl-amide gel and transferred to the nitrocellulose membrane. GAPDH antibody (1:3,000 Cell Signaling Technology, Beverly, MA, USA), rabbit anti-myostatin antibody (1:1,000; Millipore, Billerica, MA, USA), rabbit anti-PI3K (1:1,000; Cell Signaling, USA) and rabbit anti-AKT (1:1,000; Cell Signaling, USA) were used as primary antibodies. Band detection was performed using the enhanced chemiluminescence detection kit (Santa Cruz Biotechnology, Dallas, CA, USA).

### Statistical analysis

Values are shown as the mean ± standard deviation (SD) of at least three independently experiments. Differences between groups were determined by Student’s t-test, with values of *p < 0.05 and **p < 0.01 considered to be significantly different.

## Electronic supplementary material

Supplementary information
