# Supplementary material for: Mussel Inspired Polynorepinephrine Functionalized Electrospun Polycaprolactone Microfibers for Muscle Regeneration
Source: Sci Rep. 2017 Aug 15;7:8197. doi: 10.1038/s41598-017-08572-z (PMC5557809; doi:10.1038/s41598-017-08572-z)
Supplement: Supplementary file 1 — Supplementary information [file 41598_2017_8572_MOESM1_ESM.pdf]

Supporting Information

Mussel Inspired Polynorepinephrine Functionalized Electrospun Polycaprolactone Microfibers for Muscle Regeneration

Ying Liu[#], Guoqiang Zhou[#], Zhu Liu, Mengyu Guo, Xiumei Jiang, Mehmet Berat Taskin, Zhongyang Zhang, Jing Liu, Jinglong Tang, Ru Bai, Flemming Besenbacher, Menglin Chen, Chunying Chen**

Ying Liu, Mengyu Guo, Xiumei Jiang, Jing Liu, Jinglong Tang, Ru Bai, Chunying Chen
CAS Key Laboratory for Biomedical Effects of Nanomaterials and Nanosafety and CAS Center for Excellence in Nanoscience, National Center for Nanoscience and Technology (NCNST), Beijing 100190, China
Chunying Chen, chenchy@nanoctr.cn, Tel.: +86 10 82545560; fax: +86 10 62656765

Mehmet Berat Taskin, Zhongyang Zhang, Flemming Besenbacher, Menglin Chen
Interdisciplinary Nanoscience Center (iNANO), Aarhus University, DK-8000 Aarhus C, Denmark
Menglin Chen, menglin@inano.au.dk

Guoqiang Zhou, Zhu Liu
Key Laboratory of Medicinal Chemistry and Molecular Diagnosis of Ministry of Education, College of Chemistry and Environmental Science, Hebei University, Baoding, China

Ying Liu and Guoqiang Zhou contributed equally to this work.

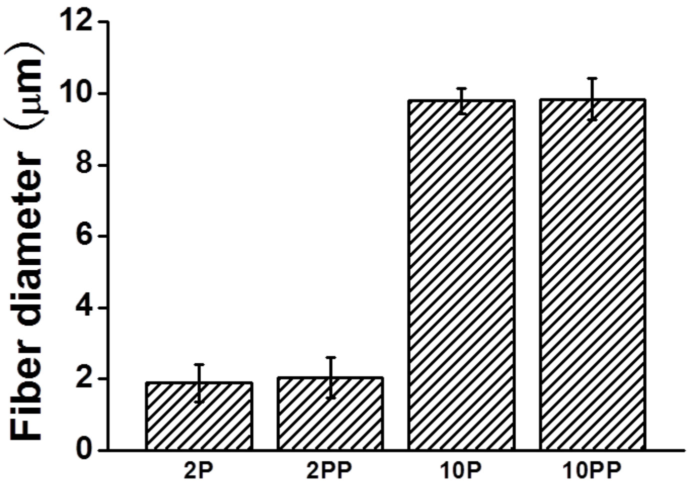

Figure S1. The diameter of PCL fibrous membranes.

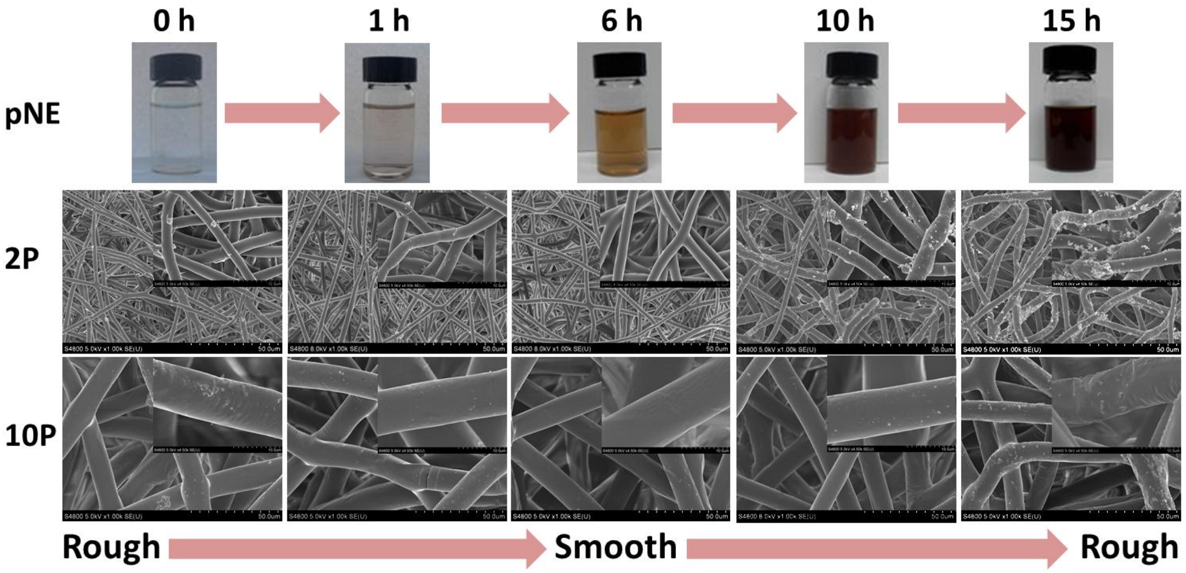

Figure S2. The process of polynorepinephrine (pNE) coating PCL fibrous membranes.

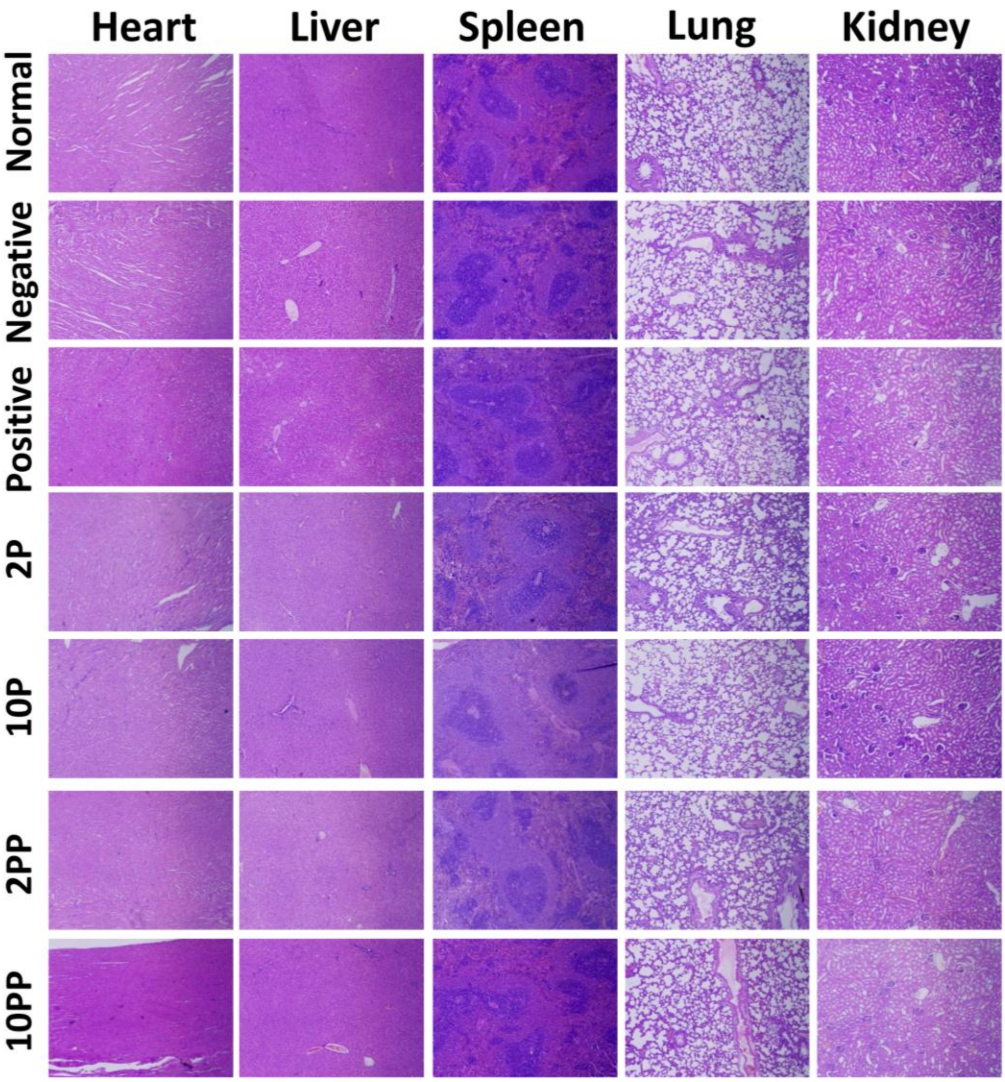

Figure S3. Histological observations of liver, spleen, lung and kidneys.

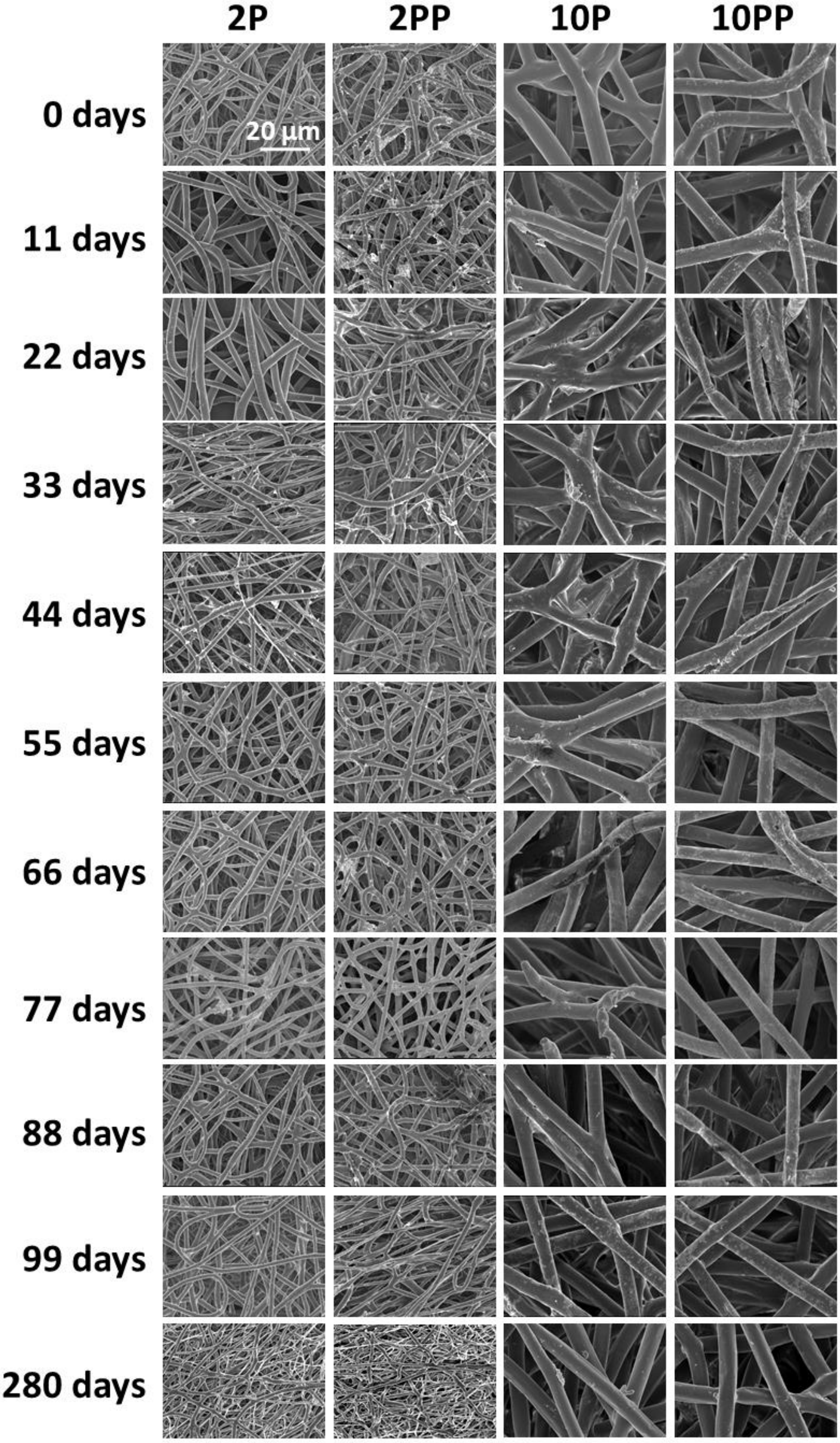

Figure S4. The degradation profile of PCL fibrous membranes *in vitro*. SEM images of PCL fibrous membranes after cultured in PBS at 37 ℃ for 11, 22, 33, 44, 55, 66, 77, 88, 99 and 280 days, respectively.

Figure 5.

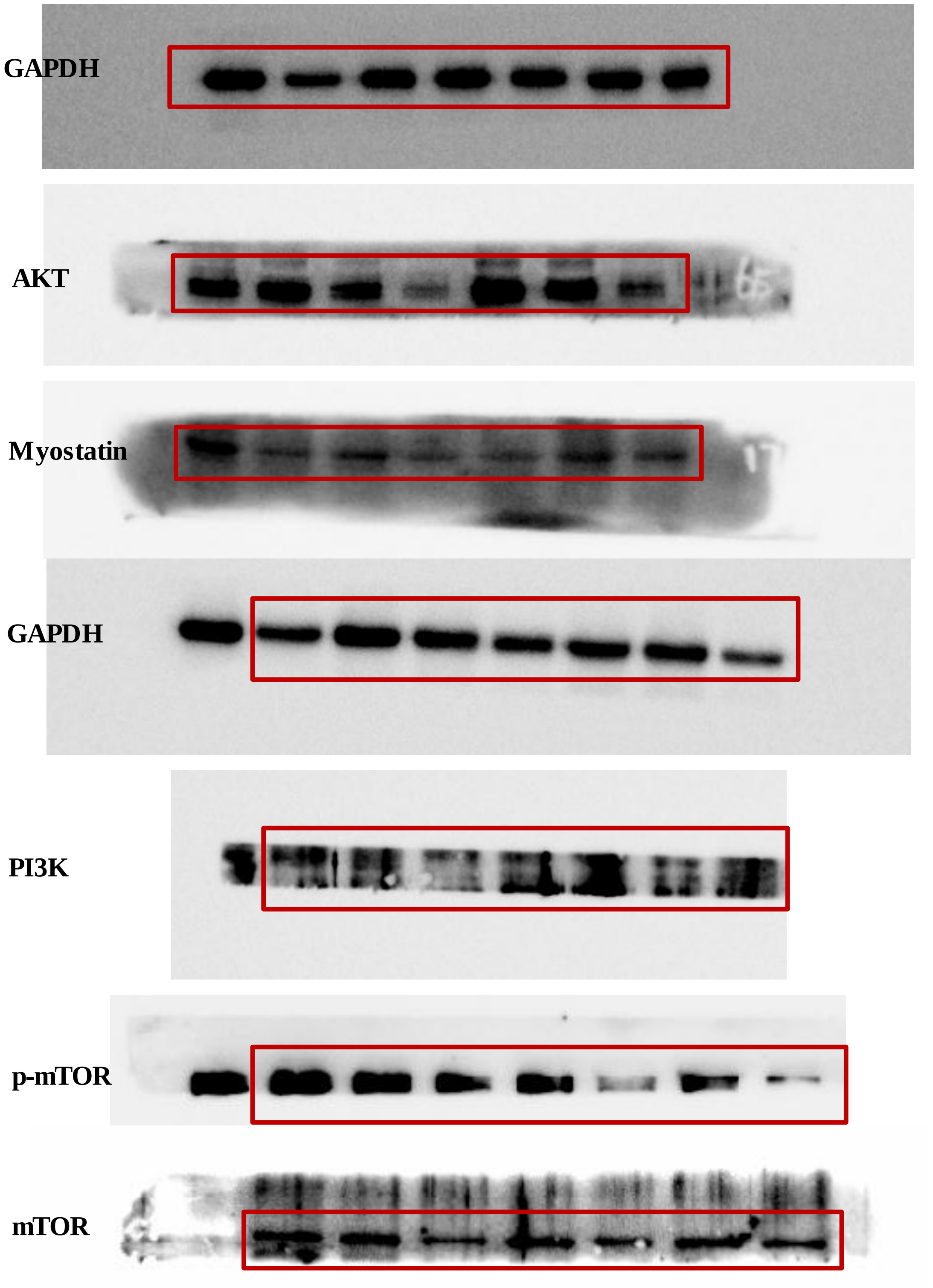

Figure S5. Uncropped western blots with indicated areas of selection.

Table S1. The body weights and coefficients of various tissues to body weight at the end of treatment

	Organ coefficients (mg/g)				
**Group**	**Body weight (g)**	**Liver**	**Spleen**	**Lung**	**Kidneys**
**Normal**	274.5 ± 5.97	40.4 ± 3.63	2.6 ± 0.33	5.2 ± 0.46	8.4 ± 0.59
**Negative**	286.3 ± 14.63	35.7 ± 1.47*	2.5 ± 0.19	4.9 ± 0.41	8.1 ± 0.58
**Positive**	275.1 ± 16.35	39.1 ± 1.23	2.6 ± 0.23	5.3 ± 0.37	7.9 ± 0.41
**2 P**	276.9 ± 16.38	36.7 ± 2.75	2.8 ± 0.68	5.1 ± 0.20	8.2 ± 0.34
**2 PP**	282.2 ± 5.87	37.0 ± 2.61	2.5 ± 0.39	5.1 ± 0.39	8.0 ± 0.66
**10 P**	274.0 ± 11.25	36.1 ± 6.14	2.6 ± 0.34	4.9 ± 0.42	8.2 ± 0.61
**10 PP**	271.5 ± 10.55	34.3 ± 1.37*	2.6 ± 0.70	4.8 ± 0.19	8.3 ± 0.34

*** Represents significant difference compared to the control group, * $p < 0.05$.**

Table S2. The blood coagulation factors

Group	PT (sec)	INR	PTA (%)	PT-R	APTT (sec)	APTT-R	TT (sec)
**Normal**	10.0 ± 0.25	0.9 ± 0.02	117.3 ± 5.03	0.9 ± 0.02	17.8 ± 1.04	0.6 ± 0.04	27.8 ± 4.09
**Negative**	9.5 ± 0.36	0.9 ± 0.04	129.3 ± 8.62	0.9 ± 0.03	17.6 ± 0.76	0.6 ± 0.02	28.3 ± 8.24
**Positive**	9.5 ± 0.45	0.9 ± 0.04	130.3 ± 11.06	0.8 ± 0.04	17.2 ± 1.04	0.6 ± 0.03	27.1 ± 7.49
**2 P**	9.6 ± 0.15	0.9 ± 0.02	130.3 ± 4.04	0.8 ± 0.02	17.8 ± 1.25	0.6 ± 0.05	25.3 ± 3.84
**2 PP**	9.7 ± 0.15	0.9 ± 0.02	123.3 ± 3.05	0.9 ± 0.01	17.9 ± 0.62	0.7 ± 0.02	30.7 ± 2.21
**10 P**	9.3 ± 0.40	0.8 ± 0.04	133.3 ± 10.69	0.8 ± 0.04	19.5 ± 1.35	0.7 ± 0.05	25.9 ± 1.65
**10 PP**	9.9 ± 0.36	0.9 ± 0.03	120.0 ± 7.21	0.9 ± 0.03	20.4 ± 0.85	0.7 ± 0.03	27.6 ± 1.29

Table S3. Complete blood counts

Group	WBC (10^9/L)	RBC (10^{12}/L)	HGB (g/L)	NEUT (%)	LY (%)	MONO (%)
**Normal**	7.5 ±3.63	6.4 ±0.33	136.7 ±3.21	11.7 ±2.27	86.4 ±2.31	2.2 ±0.32
**Negative**	2.6 ±0.29	7.0 ±1.20	133.3 ±2.55	11.6 ±3.40	84.8 ±3.69	2.0 ±1.03
**Positive**	5.4 ±2.89	7.0 ±0.37	139.7 ±13.50	17.1 ±5.82	79.9 ±5.88	2.2 ±0.76
**2 P**	4.3 ±2.55	6.7 ±0.34	126.0 ±7.21	17.4 ±5.16	79.6 ±5.95	1.7 ±0.85
**2 PP**	4.6 ±0.76	7.7 ±1.41	134.7 ±6.11	17.4 ±4.96	80.2 ±5.65	1.6 ±1.40
**10 P**	3.7 ±1.24	7.0 ±0.33	130.0 ±5.57	16.9 ±5.88	81.2 ±2.23	1.7 ±1.22
**10 PP**	2.1 ±0.63	6.9 ±0.73	129.7 ±14.57	14.9 ±4.24	82.7 ±5.43	1.8 ±0.25

Table S4. Complete blood counts (Continued)

Group	Hct (L/L)	MCV (fL)	MCH (pg)	MCHC (g/L)	RDW (%)	PLT (10^{-9}/L)	PCT (%)	MPV (fL)	PDW (fL)
**Normal**	0.4 ± 0.01	66.7 ± 3.00	21.5 ± 0.65	323.0 ± 5.57	13.9 ± 0.15	651.3 ± 34.99	0.5 ± 0.03	7.8 ± 0.06	7.4 ± 0.36
**Negative**	0.4 ± 0.06	56.8 ± 1.39*	19.1 ± 0.50**	336.7 ± 4.16*	12.4 ± 0.42*	807.3 ± 16.17**	0.6 ± 0.01*	7.7 ± 0.12	7.7 ± 0.25
**Positive**	0.4 ± 0.04	57.2 ± 2.37*	19.8 ± 0.90	326.3 ± 4.08	12.3 ± 0.31*	658.3 ± 24.25	0.6 ± 0.14	7.9 ± 0.20	7.9 ± 0.35
**2 P**	0.4 ± 0.03	60.4 ± 2.57	20.9 ± 0.62	321.3 ± 3.05	13.6 ± 0.71	692.7 ± 32.75	0.6 ± 0.03	7.9 ± 0.25	7.7 ± 0.36
**2 PP**	0.4 ± 0.01	52.9 ± 8.05	17.7 ± 2.40	336.0 ± 9.54	14.4 ± 2.70	663.3 ± 32.10	0.5 ± 0.03	7.3 ± 0.28	7.1 ± 0.23
**10 P**	0.4 ± 0.02	63.0 ± 0.26	20.6 ± 0.20	314.7 ± 3.79	14.0 ± 0.36	719.3 ± 54.88	0.5 ± 0.08	7.7 ± 0.10	7.5 ± 0.17
**10 PP**	0.4 ± 0.03	59.7 ± 5.37	21.7 ± 0.15	319.3 ± 10.12	13.8 ± 0.53	650.0 ± 38.28	0.4 ± 0.26	7.7 ± 0.06	7.5 ± 0.40

*** and ** represent significant difference compared to the normal control group, *$p < 0.05$ and **$p < 0.01$, respectively.**

Table S5. Blood biochemical analyses

Group	BUN (mmol/L)	CR (mmol/L)	ALT (U/L)	AST (U/L)	LDH (U/L)	CK (U/L)	TP (g/L)	ALB (g/L)
**Normal**	11.8 ± 0.42	12.3 ± 0.58	49.3 ± 5.70	134.0 ± 5.71	588.3 ± 24.11	602.7 ± 52.26	55.9 ± 2.08	33.6 ± 0.87
**Negative**	14.4 ± 0.85**	11.3 ± 3.21	46.3 ± 4.04	175.3 ± 10.69*	1947.7 ± 59.21**	641.3 ± 70.51	56.1 ± 2.73	32.1 ± 0.35
**Positive**	10.4 ± 1.97	11.0 ± 1.73	48.0 ± 7.00	144.7 ± 16.56	618.7 ± 39.83	615.0 ± 52.07	56.3 ± 0.74	33.2 ± 1.69
**2 P**	11.4 ± 1.59	10.0 ± 3.61	54.0 ± 7.00	139.7 ± 13.87	613.0 ± 23.14	629.7 ± 33.89	55.7 ± 2.88	33.5 ± 0.61
**2 PP**	12.1 ± 1.55	11.7 ± 1.00	48.7 ± 3.69	138.8 ± 13.35	603.3 ± 49.29	607.0 ± 42.28	54.2 ± 1.27	33.3 ± 0.31
**10 P**	11.7 ± 1.19	9.3 ± 2.31	51.2 ± 1.53	140.3 ± 7.02	622.7 ± 45.51	611.7 ± 24.18	53.6 ± 2.08	33.5 ± 1.13
**10 PP**	10.9 ± 0.55	9.67 ± 1.53	62.0 ± 9.00	138.7 ± 12.37	614.3 ± 30.22	597.7 ± 25.46	53.1 ± 2.29	33.6 ± 1.25

*** and ** represent significant difference compared to the normal control group, *$p < 0.05$ and **$p < 0.01$, respectively.**
